# Discovering New Substrates of a UDP-Glycosyltransferase with a High-Throughput Method

**DOI:** 10.3390/ijms25052725

**Published:** 2024-02-27

**Authors:** Mary C. L. Lethe, Dinh Bui, Ming Hu, Xiaoqiang Wang, Rashim Singh, Clement T. Y. Chan

**Affiliations:** 1 Department of Biomedical Engineering, College of Engineering, University of North Texas, 3940 N Elm Street, Denton, TX 76207, USA; marycarolinlethe@my.unt.edu; 2 Department of Pharmacological and Pharmaceutical Sciences, College of Pharmacy, University of Houston, 4349 Martin Luther King Boulevard, Houston, TX 77204, USA; 2dinhbt@gmail.com (D.B.); mhu@uh.edu (M.H.); rashim.singh@sanarentero.com (R.S.); 3 Department of Biological Sciences, College of Science, University of North Texas, 1155 Union Circle #305220, Denton, TX 76203, USA; xiaoqiang.wang@unt.edu; 4 Sanarentero LLC, 514 N. Elder Grove Drive, Pearland, TX 77584, USA; 5 BioDiscovery Institute, University of North Texas, 1155 Union Circle #305220, Denton, TX 76203, USA

**Keywords:** xenobiotics metabolism, high-throughput screening, BPA, SN-38, UDP-glycosyltransferase

## Abstract

UDP-glycosyltransferases (UGTs) form a large enzyme family that is found in a wide range of organisms. These enzymes are known for accepting a wide variety of substrates, and they derivatize xenobiotics and metabolites for detoxification. However, most UGT homologs have not been well characterized, and their potential for biomedical and environmental applications is underexplored. In this work, we have used a fluorescent assay for screening substrates of a plant UGT homolog by monitoring the formation of UDP. We optimized the assay such that it could be used for high-throughput screening of substrates of the *Medicago truncatula* UGT enzyme, UGT71G1, and our results show that 34 of the 159 screened compound samples are potential substrates. With an LC–MS/MS method, we confirmed that three of these candidates indeed were glycosylated by UGT71G1, which includes bisphenol A (BPA) and 7-Ethyl-10-hydroxycamptothecin (SN-38); derivatization of these toxic compounds can lead to new environmental and medical applications. This work suggests that UGT homologs may recognize a substrate profile that is much broader than previously anticipated. Additionally, it demonstrates that this screening method provides a new means to study UDP-glycosyltransferases, facilitating the use of these enzymes to tackle a wide range of problems.

## 1. Introduction

Glycosyltransferases are present in a broad range of organisms, including animals, plants, and microbes [1,2,3]. These enzymes transfer glycosyl groups from activated sugars, such as uridine-5′-diphosphate-glucose (UDP-glucose), to target substrates. Substrates of glycosyltransferases are highly diverse and can be polysaccharides, monosaccharides, lipids, alkaloids, antibiotics, plant hormones, nucleic acids, proteins, and toxic xenobiotics [4,5,6,7]. Some UDP glycosyltransferase (UGT) enzymes are known for derivatizing toxic molecules that cause various diseases. For instance, deoxynivalenol is a mycotoxin that is present in rotten grains, such as rice and wheat, which leads to a variety of symptoms, including nausea, vomiting, diarrhea, and fever [8,9]. Several reports show that this toxin can be glycosylated by several UGT homologs [10,11,12]. Some other toxins that can be modified by UGTs include zearalenone [13], nivalenol [14], and alternariol [15]. For metabolic engineering, UGTs were harnessed to create biosynthetic pathways in microbes to create drug candidates [16,17,18]. On the other hand, some UGT genes were explored for engineering crops as they can improve tolerance to drought [19,20], pathogens [21,22], and extreme temperatures [23,24]. These examples demonstrate the broad utility of UGT family enzymes for medicinal and agricultural applications. Therefore, researchers have put a huge effort into studying the protein superfamily of glycosyltransferases and exploring the use of these enzymes [25,26,27,28,29,30,31,32].

The UGT superfamily has great potential for biotechnology developments, and over 80,000 family members have been identified [33]. However, only a tiny fraction of these UGT homologs have been characterized. In some studies, the substrates of UDP glycosyltransferase (UGT) homologs were not identified, but genetic experiments showed that they provided benefits to the plants, such as improved tolerance to drought [23,34], herbicides [27,28,35], and infection [36,37]. A vast number of UGT homologs are waiting to be explored for applications in many fields. 

One main challenge in studying UGTs is the time and labor required to characterize their substrate specificity. Conventionally, liquid chromatography approaches are used to isolate compounds after a glycosyltransferase reaction, such that the presence of glycosylated products can be detected [38,39]. To test different substrate candidates, adjustments to the chromatographic method may be required to observe the product, which can consume a substantial amount of time and labor. In many cases, mass spectrometric techniques are also required to confirm the products [40]. These limitations highly restrict the characterization of substrate profiles for these enzymes.

In this study, we screened for substrates of a plant UGT by coupling a commercially available UDP detection assay with the UGT enzymatic activities, which transfer the glucosyl moiety from UDP-glucose to an acceptor molecule. This method is based on detecting the formation of UDP molecules when the glycosyl moiety of UDP-glucose dissociates from UDP during the glycosylation reaction. The ease of receiving a readout allows the use of this method to perform high-throughput screening for substrates of a plant UGT homology, UGT71G1, from *Medicago truncatula*. Previous studies showed that UGT71G1 glycosylates multiple hydroxyl groups in various natural products, such as genistein and biochanin A [41]. Additionally, UGT71G1 is a cytosolic enzyme that remains active when expressed in bacterial cells [41,42]. These properties render this UGT homolog to have great potential in metabolic applications.

With this high-throughput assay, we screened 159 samples for UGT71G1 activities, which include drugs and metabolites, natural products, and environmental/dietary toxins. Among positive candidates in our screening results, we used an LC–MS/MS method to confirm glycosylated products from three new substrates with potential medical and environmental applications, including an environmental pollutant from plastic, bisphenol A (BPA) [43,44], and an active and toxic metabolite of the anticancer drug irinotecan, 7-Ethyl-10-hydroxycamptothecin (SN-38) [45,46]. We specifically selected to validate our screening results for these compounds because a new method for derivatizing these toxicants may be valuable, and these discoveries may facilitate the use of this UGT71G1 to develop engineered organisms for removing these toxic compounds, which represents a new way to improve human health. Together, these results show that the substrate profiles of some UGT homologs can be much broader than what we have known. Our work supports the use of this high-throughput method for discovering new substrates of UGT homologs, and it highlights the potential of UGT71G1 for a wide range of applications.

## 2. Results

### 2.1. High-Throughput Fluorescent Assay to Detect UDP-Glycosyltransferase Activities

**Figure 1:**
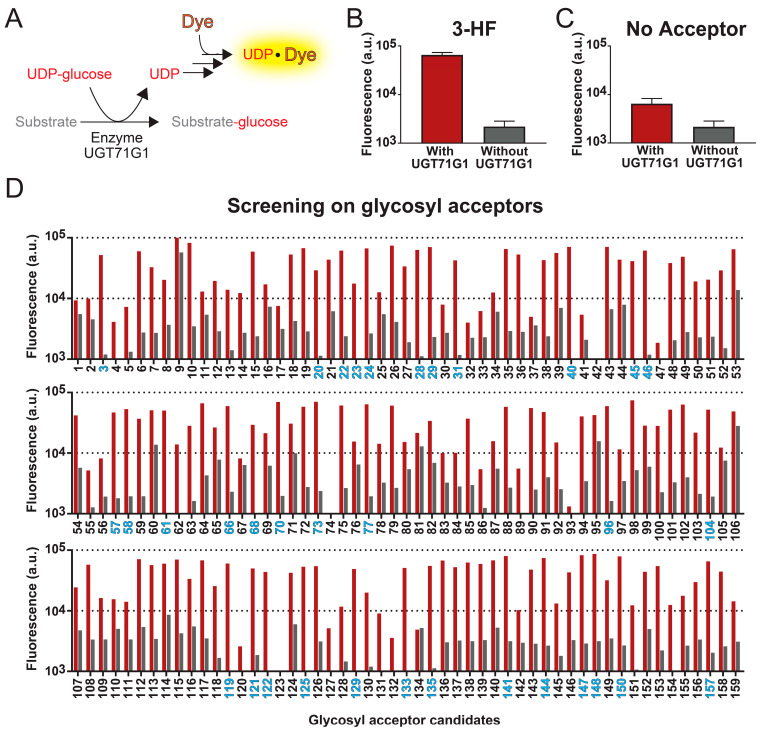
High-throughput fluorescent assay for screening substrates of UGT enzymes. (**A**) Reactions of the UGT assay. Detection of UDP-glycosyltransferase activities is based on the formation of a UDP-dye complex, which increases the fluorescence of the sample. (**B**) With this assay, we characterized the enzymatic activity of UGT71G1 with 3-hydroflavone as the substrate (red bar). As a control experiment, the assay was also performed with the sample conditions except without the enzyme UGT71G1 (grey bar). (**C**) As another negative control, the assay was performed in the absence of a glycosyl acceptor, aiming to determine whether the fluorescence signal increase is due to the UGT activities or not. Data in panels B and C are from six biological replicates ± S.D. (**D**) This method was then used to test 159 compounds. For each compound, fluorescence signal levels were compared in an assay with (red bar) and without (grey bar) the enzyme UGT71G1. A compound is considered a good substrate for UGT71G1 when the ratio of fluorescence with and without UGT71G1 is above 25. The number of good substrates is colored blue; a list of these compounds is in Supplementary Table S1.

For a high-through method to robustly detect UGT activities with different glycosyl acceptors, it is necessary to target a change that is independent of the acceptor molecule. UDP-glycosyltransferases take UDP-glucose as a co-substrate; while the glucosyl moiety is transferred to the acceptor molecule, UDP is released as a by-product (Figure 1A). Thus, UGT activities can be detected by the rise in UDP levels in the reaction mixture. In the method to monitor an increase in UDP concentration, the UGT71G1 enzymatic reaction was coupled to the commercial UDP assay kit, in which the released UDP leads to an increase in fluorescent signal. By monitoring the increase in this fluorescence intensity, the formation of UDP and UGT enzymatic activities can be detected.

With this high-throughput method, we tested 159 samples as glycosyl acceptors, which include 143 compounds and some replicates of these candidates from different sources. The xenobiotic, 3-hydroxyflavone (3HF), is one of the candidates that generated a significant increase in fluorescence intensity when tested with the abovementioned assay. To determine whether the rise in fluorescence intensity is due to UGT activities, we also performed the assay in the absence of enzyme UGT71G1, evaluating the background fluorescent signals from the compound sample. As shown in Figure 1B, the presence of both 3HF and UGT71G1 led to a 30-fold increase in fluorescent signal compared to the condition without UGT71G1. Similarly, we evaluate the detection method in the absence of a glycosyl acceptor (Figure 1C). While we did not expect a change in fluorescent signal in the absence of glycosyl acceptor, the presence of enzyme UGT71G1 alone caused a 3-fold increase in the signal, which suggests that our isolated UGT71G1 contains contaminating hydrolases that non-specifically hydrolyze UDP-glucose, generating a basal level of UDP.

Among the 143 compounds that we screened with the high-throughput assay, they were selected based on their structural similarity to other known substrates of human UGT enzymes. This compound library is shown in Supplementary Table S1. Each compound was assayed in both the presence and absence of the enzyme UGT71G1 (Figure 1D). To test the robustness of this assay, 13 compounds were run in duplicate, and 1 compound (3-hydroxy-6-methoxyflavone; compound **24**) was run four times; replicates of the same compound were from different stocks.

Among those 144 compounds, 31 of them are strong candidates as glycosyl acceptors, and they generated an over 25-fold increase in fluorescence intensity with UGT71G1 (Figure 1D and Supplementary Table S2). Most of these candidates are natural plant flavonoids (including seven flavones, eight flavonols, two flavonones, three isoflavones, two chalcones, and one anthocyanin), and they are potentially among those natural substrates of UGT71G1. A few other strong candidates are xenobiotics, including the drug metabolite SN-38 (compound **58**) and the environmental toxin bisphenol A (BPA; compound **135**). Both of these two candidates are cytotoxic, so they can be targets for health-related applications. SN-38 is the active metabolite of a chemotherapeutic agent, irinotecan; among patients that administer irinotecan, SN-38 is excreted into the intestines via bile, which leads to 25% of these patients experiencing diarrhea [47]. For BPA, it is broadly used in the manufacturing of plastics, which leads to its spread in the environment. Many studies have shown that BPA affects the development of the brain and prostate glands [48,49]. Therefore, using UGT71G1 to derivatize these compounds may provide an efficient means to reduce human exposure to them. Other strong candidates include coumarin (1), smaller phenols (3), polyphenol (1), anthraquinone (1), and the microbial metabolite of proanthocyanidin (1). Interestingly, substrates of UGT71G1 ranged from compounds with a single hydroxy group in structure to multiple hydroxy groups (e.g., cyanidin, isorhamnetin) and could be glycosylated at multiple hydroxyl positions (only confirmed with one compound, diglucose of bisphenol A; Figure 1C). However, there was no obvious structure–activity relationship that could be drawn from the results. 

Among the rest of the glycosyl acceptor candidates, most of them produced low fluorescence signals in both the presence and absence of UGT71G1, implying that they do not serve as effective substrates for this enzyme in this assay condition. However, several compounds led to significant levels of fluorescence even in the absence of UGT71G1, suggesting that they either directly cause the release of UDP or interact with the fluorescent dye. The most representative compounds in this category include sorafenib (compound **9**), 7,2′-dihydroxy flavone (compound **53**), 5,4′-dihydroxyflavone (compound **60**), and 7-hydroxy-4′-methoxy-flavone (compound **106**). As a result, our method cannot accurately assess the efficiency of UGT activities for these candidates.

### 2.2. Confirmation of UDP-Glycosyltransferase Activities with LC–MS/MS

**Figure 2:**
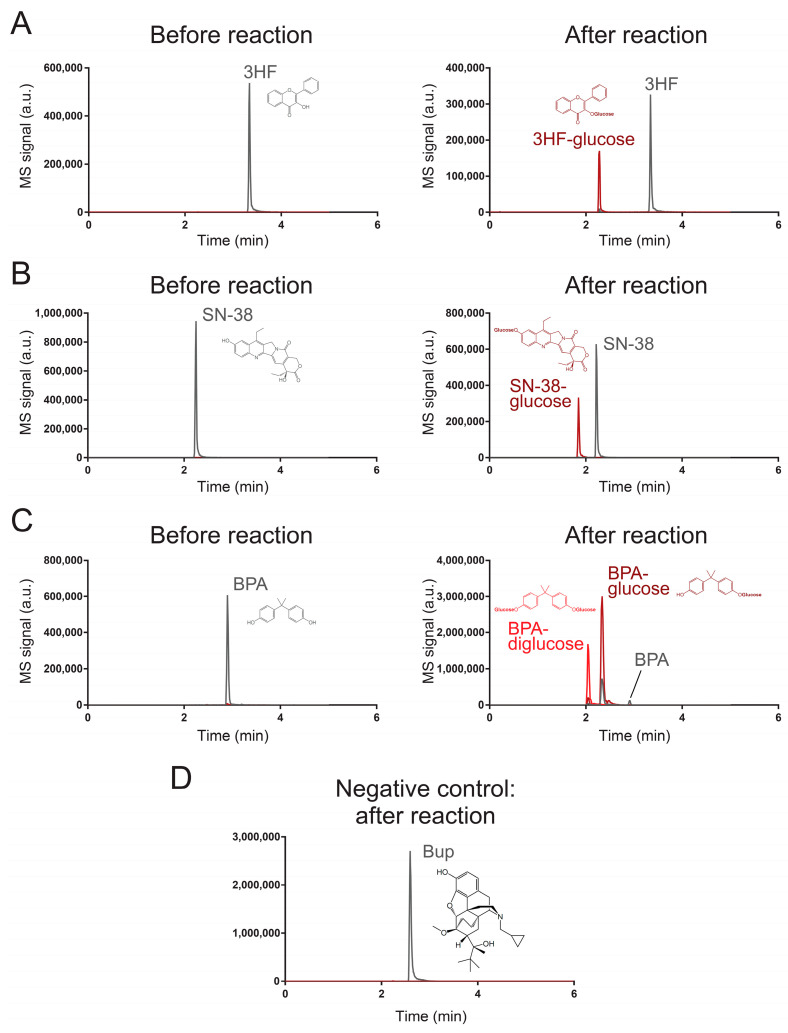
Characterization of UGT71G1-catalyzed reactions with an LC–MS/MS method. These chromatograms characterize the substrates and products of UGT71G1 reacting with (**A**) 3HF, (**B**) SN-38, (**C**) PBA, and (**D**) Bup. MS/MS transitions for monitoring each substrate and product species are listed in Table 1. Tracing lines for monitoring substrates are colored gray, and those for products are colored red. In the case of PBA, two products are colored bright red and dark red. Transitions for monitoring substrates may be sensitive to some products; this is because, during ionization by the nebulizer, a product can be fragmentized to become an ion that is the same as the substrate. For Bup, it serves as a negative control, and no substantial signal for potential products was detected.

To validate our results from the high-throughput fluorescent assay, we used a liquid chromatography-coupled tandem mass spectrometric (LC–MS/MS) method to monitor the reaction of UGT71G1 with 3-hydroxyflavone, bisphenol A, and SN-38. Table 1 shows the transitions that are used to monitor these substrates and their products in our LC–MS/MS method. Analytical standards were used to confirm the elution of three xenobiotic substrates, which formed those peaks labeled in Figure 2. After reaction with the enzyme UGT71G1 in the presence of co-substrate UDP-glucose, at least one new peak was formed in the LC–MS/MS analysis of the three substrate samples, and the *m*/*z* values match the assigned structure of the *O*-glucose conjugate(s) as shown.

For 3-hydroxyflavone (Figure 2A), it eluted at 3.34 min, and the metabolite peak eluted at 2.28 min with a parental ion *m*/*z* value of 401, which is the expected *m/z* value of a 3-hydroxyflavone with glucose on its original hydroxyl group. After collision-induced dissociation (MS/MS), the major fragment has an *m*/*z* value the same as that of 3-hydroxyflavone (Q1/Q3 transition masses 401/239). Similarly, SN-38 (Figure 2B) eluted at 2.22 min and its glucose derivative eluted at 1.85 min with expected *m*/*z* for the parental ion and the fragmented ion (the fragmented ion’s *m*/*z* is the same as that of SN-38; Q1/Q3 transition masses 555/393). For BPA (Figure 2C), the reaction catalyzed by UGT71G1 led to two products. The major product had a parental ion with a *m*/*z* value as a BPA with one hydroxyl group glycosylated (elution time was 2.32 min) (Q1/Q3 transition masses 389/227). The minor product was expected to be a BPA with both of its hydroxyl groups glycosylated (Q1/Q3 transition masses 551/227) and eluted earlier than the monoglycosylated product (2.04 min). Multiple Reaction Monitoring (MRM) chromatograms are shown for 3HF (Figure S1), 3HF-glucose (Figure S2), SN-38 (Figure S3), SN-38-glucose (Figure S4), BPA (Figure S5), BPA-glucose (Figure S6), and BPA-diglucose (Figure S7). These results strongly support the idea that the three analyzed xenobiotics are substrates of UGT71G1 for glycosylation.

Next, we tested whether the screening assay could also identify candidates that are not efficient substrates of UGT71G1 or not. For this purpose, we used the LC/MS–MS method to characterize the enzymatic reaction with buprenorphine (Bup; compound **152**). This compound was selected because the screening assay shows that Bup generated an 8.8-fold increase in fluorescence intensity with UGT71G1 (Figure 1D), which is lower than our selected threshold of a 25-fold increase. We aimed to test if this level of signal change could indicate a potential substrate. As shown in Figure 2D, the LC/MS–MS analysis detected the presence of Bup (Q1/Q3 transition masses 468/414), but it did not detect any substantial peak with the expected ion transition for the glycosylated Bup (Q1/Q3 transition masses 630/468). These results confirm that Bup is not an efficient substrate for UGT71G1. 

## 3. Discussion

In this work, we have harnessed a commercial UDP detection kit for high-throughput screening of substrates of uridine-glycosyltransferases. Previous studies have developed a range of methods for monitoring glycosylation. For instance, radiochemical assays were developed that are based on detecting the transfer of radiolabeled sugar moiety from the donor to the acceptor by scintillation counting [50,51]. This method is highly sensitive, but the use of radioactive materials can be undesirable. Another strategy is to monitor changes in pH [50,52,53]. During glycosylation, UDP-glucose is hydrolyzed and protons are generated, which leads to a decrease in pH. By using pH-sensitive dyes, previous studies have observed glycosylation and used this method to initiate the screening of substrates. However, the change in pH can be affected by the buffer system as well as the substrate and product molecules in the assay. The low stability of these pH-based assays limits their use in high-throughput screening of substrate profiles. 

The assay that was used in this study has avoided some limitations in the abovementioned methods. As this assay is based on detecting the release of UDP from the glucose moiety, it is independent of the glycosyl acceptor, which allows its utilization for screening a wide range of substrate candidates. Additionally, UDP is a stable molecule during the period and conditions of our assay, which allows robust detection of its formation. Compared to the traditional method for characterizing UGT activities that require LC–MS/MS techniques, this fluorescent assay requires less time, labor, and instrumentation costs, which facilitates the screening of an extensive set of compounds for studying substrate specificity.

With all these advantages, this assay has limitations. It only indirectly detects a reaction that generates UDP and cannot provide any structural information about products. Additionally, this method is not feasible for testing some compounds; as described, some candidates, such as sorafenib (compound **9**), 7,2′-dihydroxy flavone (compound **53**), 5,4′-dihydroxyflavone (compound **60**), and 7-hydroxy-4′-methoxy-flavone (compound **106**), led to high background levels of fluorescent signal, which hindered the observation of the reaction for UDP formation. It is highly possible that some other candidates can inhibit reactions to the assay, rendering a loss of fluorescent signal even when UDP is formed. Therefore, additional techniques, such as LC–MS/MS, are still required to validate the results of our high-throughput assay.

With this method, we have discovered a set of potential substrates for a plant UGT homolog, UGT71G1, and we have validated three of them, including 3HF, BPA, and SN-38. 3HF is a synthetic flavonoid that is not found naturally [54], and thus, the glycosylation of this compound may not provide any substantial benefits. In contrast, a robust method for derivatizing BPA and SN-38 can lead to significant biomedical applications as these two compounds are related to toxicity-induced health issues. BPA can be released from polycarbonate plastics, and its presence is now ubiquitous in the environment [48]. Recent studies support the idea that BPA may induce carcinogenesis and mutagenesis [55,56]. This xenobiotic may also generate adverse health effects on brain development, potentially affecting children’s behavior [57]. SN-38 is a cytotoxic metabolite of the chemotherapeutic agent irinotecan; when it is excreted to the gut via bile, it can lead to a range of gastrointestinal side effects, such as severe delayed onset diarrhea [58]. If the glycosylation of these xenobiotics reduces their toxicity, it can provide a new means to improve human health.

The plant UGT homology, UGT71G1, has the potential for biomedical and environmental applications. Many well-studied UGT homologs, such as the human UGT1A1, are membrane proteins that are difficult to express and purify. For the plant UGT71G1, UGT71G1 is a highly soluble cytosolic protein, and it is active when expressed in bacteria. These properties facilitate its use in many settings for derivatizing target species, such as in the digestive system or natural environments. In the near future, our high-throughput method can be used to screen an expanded set of candidates, identifying more substrates of UGT71G1 that can be detoxified by glycosylation.

Additionally, our screening method can be applied to a broad range of UDP-glycosyltransferases to characterize their substrate profiles. Based on the UniProt database, over 40,000 UDP-glycosyltransferases have been documented, with about 400 of them having already been verified on the protein level, and this list is continually expanding [59,60,61]. There is a rise in the need for characterizing substrate profiles of UGTs so that these enzymes can be harnessed for biotechnology applications. Our high-throughput assay provides a means to fulfill this need.

## 4. Materials and Methods

Materials. *E. coli BL21 (DE3)* cells containing *pET28a-UGT71G1* were prepared in previous studies [38]. LB medium broth, inducers, and antibiotics were obtained from VWR (Radnor, PA, USA). All drugs and chemicals were sourced from Sigma-Aldrich (Saint Loise, MO, USA), INDOFINE Chemical Company (Hillsborough Township, NJ, USA), or Toronto Research Chemicals (Toronto, ON, Canada). The MS-grade water, methanol, and acetonitrile were obtained from EMD (Gibbstown, NJ, USA).

Protein expression and purification of UGT71G1. The enzyme UGT71G1 was expressed in *Escherichia coli* strain BL21 (DE3) and purified as described previously with some modifications [38]. Briefly, *E. coli* BL21(DE3) cells transformed with the plasmid pET28a-UGT71G1 were grown at 37 °C in Luria–Bertani medium containing 50 mg/mL kanamycin until OD600 nm reached 0.6 to 0.8. The UGT71G1 protein expression was induced with 0.5 mM isopropyl 1-thio-β-galactopyranoside overnight at 16 °C. Cells were pelleted and resuspended in lysis buffer (50 mM Tris-HCl, pH 8.0, 500 mM NaCl, 10 mM imidazole, and 10 mM β-mercaptoethanol) and lysed by sonication on ice. After centrifugation at 12,000 rpm at 4 °C for 20 min, Ni^2+^-NTA agarose was added to the supernatant containing the target proteins. After incubation for 40 to 60 min, the mixture was transferred into a disposable column and washed extensively with lysis buffer (~100 column volumes). The His-tagged proteins were eluted with elution buffer (50 mM Tris-HCl, pH 8.0, 500 mM NaCl, 250 mM imidazole, and 10 mM β-mercaptoethanol). The protein was further purified on a Superdex-200 gel filtration column (GE Healthcare; Chicago, IL, USA) and concentrated to ~6 mg/mL in 10 mM NaCl, 10 mM Tris-HCl, pH 7.5, and 5 mM β-mercaptoethanol.

High-throughput fluorescent assay for screening candidates of UGT glycosylation. The characterization of UDP formation involved the MicroMolar UDP assay kit (ProFoldin; Hudson, MA, USA) [62]. For each reaction, reagents used from this kit include 3 µL of 10× buffer, 0.3 µL of 100× MUD reagent 1, and 0.3 µL of 100× MUD reagent 2. These reagents were mixed with 6 µg of purified enzyme UGT71G1 (this enzyme was not added for each negative control), the substrate candidate (final concentration ranging from 0.8 to 160 µM based on substrate solubility), UDP-glucose (50 µM), and ultrapure water to reach a final volume of 30 µL. The reaction mixture was incubated at 37 °C for 45 min on a 384-well microtiter plate. Each sample was then mixed with 30 µL of 1× fluorescence dye from the MicroMolar UDP assay kit, and fluorescence was measured immediately with a BioTek Synergy H1M fluorescence microplate reader at excitation 485 nm and emission 535 nm.

Liquid chromatography-coupled mass spectrometric analysis of substrates and products. The glycosylation reaction for plant UGT71G1 enzyme extracted from *E. coli* BL21(DE3) cells containing UGT71G1-pET28a was performed similarly to the glucuronidation reaction as those published previously [63] with minor modifications. Briefly, the enzyme UGT71G1 (final concentration 0.05–0.5 µg/mL) was mixed with UDP-glucose (50 μM) and substrate (final concentration 10 μM) in 50 mM potassium phosphate buffer (pH 7.4) to reach a final volume of 200 µL. This reaction mixture was incubated at 37 °C and at various time points for each substrate to obtain the highest product-to-substrate ratio: 2 h for 3HF, 4 h for SN-38 and Bup, and 24 h for BPA; reactions were ended with the addition of 200 µL methanol. The samples were centrifuged at 14,000 rpm for 15 min, and the supernatant was subjected to a UPLC–MS/MS system for analysis after appropriate dilution with a 50% MeOH solution.

LC–MS/MS analysis was performed with an ExionLC™ UHPLC system coupled with an API 5500 Q-Trap triple quadrupole mass spectrometer. A BEH C18 column (50 mm × 2.1 mm I.D., 1.7 µm, Waters, Milford, MA, USA) was used for liquid chromatography. For the mobile phase, mobile phase A (MPA) was 0.1% formic acid in water and mobile phase B (MPB) was 100% acetonitrile; the flow rate was 0.45 mL/min with a column temperature of 45 °C. An amount of 10 µL sample was injected for each analysis, and it was eluted with the following mobile phase profile: 0–0.5 min, 5–10% MPB, 0.5–1.5 min, 10–40% MPB, 1.5–4.0 min, 40–90% MPB, 4.0–4.4 min, 90% MPB, 4.4–4.6 min, 90–5% MPB, 4.6–5.0 min, 5% MPB. Gradient mobile phase for mycophenolic acid and metabolites: 0–1.0 min, 5–20% MPB, 1.0–1.5 min, 20% MPB, 1.5–4.0 min, 20–40% MPB, 4.0–5.0 min, 40–90% MPB, 5.0–5.5 min, 90–5% MPB, 5.5–6.0 min, 5% MPB.

## 5. Conclusions

In summary, our study has demonstrated the use of a platform to survey the substrate profiles of UGTs. We have used it to discover some new substrates for a plant UGT homolog. These results provide confidence that we will discover many novel substrates for a wide range of UGTs.

## Figures and Tables

**Table 1:** Transitions in tandem mass spectrometric method for monitoring UGT71G1 activities.

Compound	Parental Ion Q1 (*m*/*z*)	Fragmented Ion Q3 (*m*/*z*)	DP ^1^ (V)	CE ^2^ (V)	CXP ^3^ (V)
3HF	239	165	59	42	21
3HF-Glucose	401	239	59	42	21
SN-38	393	349	5	33	25
SN-38-Glucose	555	393	5	33	25
BPA	227	133	−157	−38	−11
BPA-Glucose	389	227	−157	−38	−11
BPA-Diglucose	551	227	−157	−38	−11
Bup	468	414	87	47	13
Bup-Glucose	630	468	106	53	13

^1^ DP: declustering potential; ^2^ CE: collision energy; ^3^ CXP: collision cell exit potential. These machine settings affect fragment compositions of MS/MS analyses.

## Data Availability

The original contributions presented in the study are included in the article/Supplementary Materials; further inquiries can be directed to the corresponding author.

## Supplementary Materials

The following supporting information can be downloaded at: https://www.mdpi.com/article/10.3390/ijms25052725/s1
