# Supplementary material for: Discovering New Substrates of a UDP-Glycosyltransferase with a High-Throughput Method
Source: Int J Mol Sci. 2024 Feb 27;25(5):2725. doi: 10.3390/ijms25052725 (PMC10931590; doi:10.3390/ijms25052725)
Supplement: Supplementary file 1 [file ijms-25-02725-s001.zip › Supplementary Figures.pdf]

Supplementary Information

Discovering New Substrates of a UDP-Glycosyltransferase with a High-Throughput Method

Mary C. L. Lethe [1], Dinh Bui [2], Ming Hu [2], Xiaoqiang Wang [3], Rashim Singh [2,4] and Clement T. Y. Chan [1,5,*]

[1] Department of Biomedical Engineering, College of Engineering, University of North Texas, 3940 N Elm Street, Denton, TX 76207, USA
[2] Department of Pharmacological and Pharmaceutical Sciences, College of Pharmacy, University of Houston, 4349 Martin Luther King Boulevard, Houston, TX 77204, USA
[3] Department of Biological Sciences, College of Science, University of North Texas, 1155 Union Circle #305220, Denton, TX 76203, USA
[4] Sanarentero LLC, 514 N. Elder Grove Drive, Pearland, TX 77584, USA
[5] BioDiscovery Institute, University of North Texas, 1155 Union Circle #305220, Denton, TX 76203, USA
* Correspondence: tszyanclement.chan@unt.edu

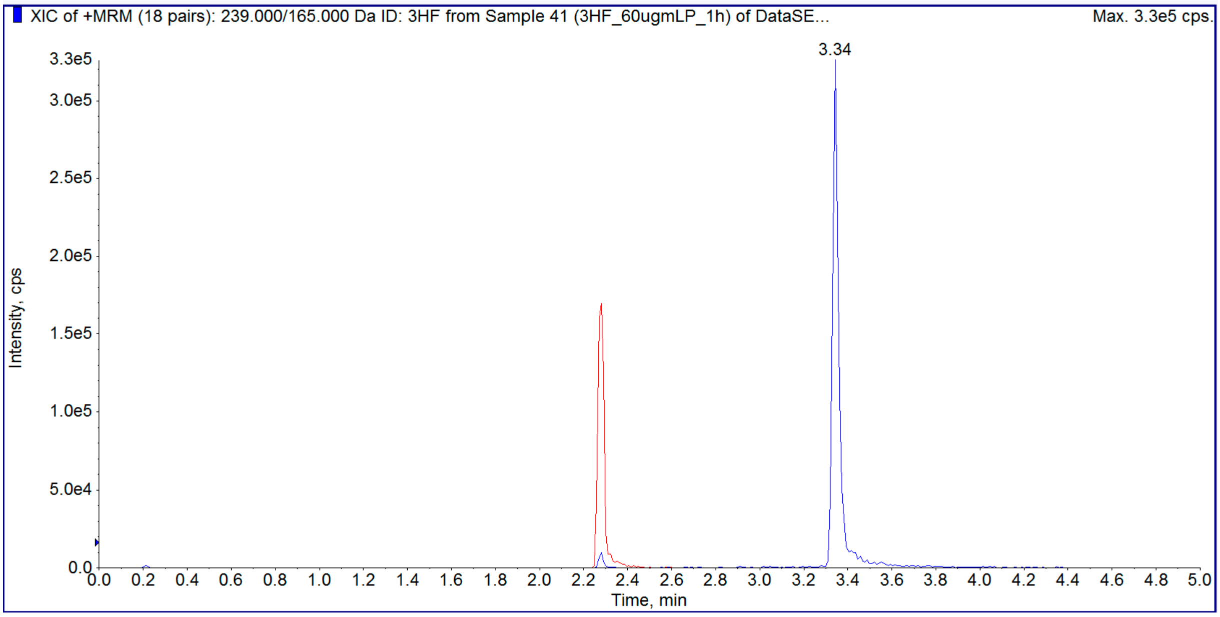

Figure S1. LC-MS Chromatogram of 3Hydroxyflavone (3HF) and its glucose metabolite (Transition 239/165).

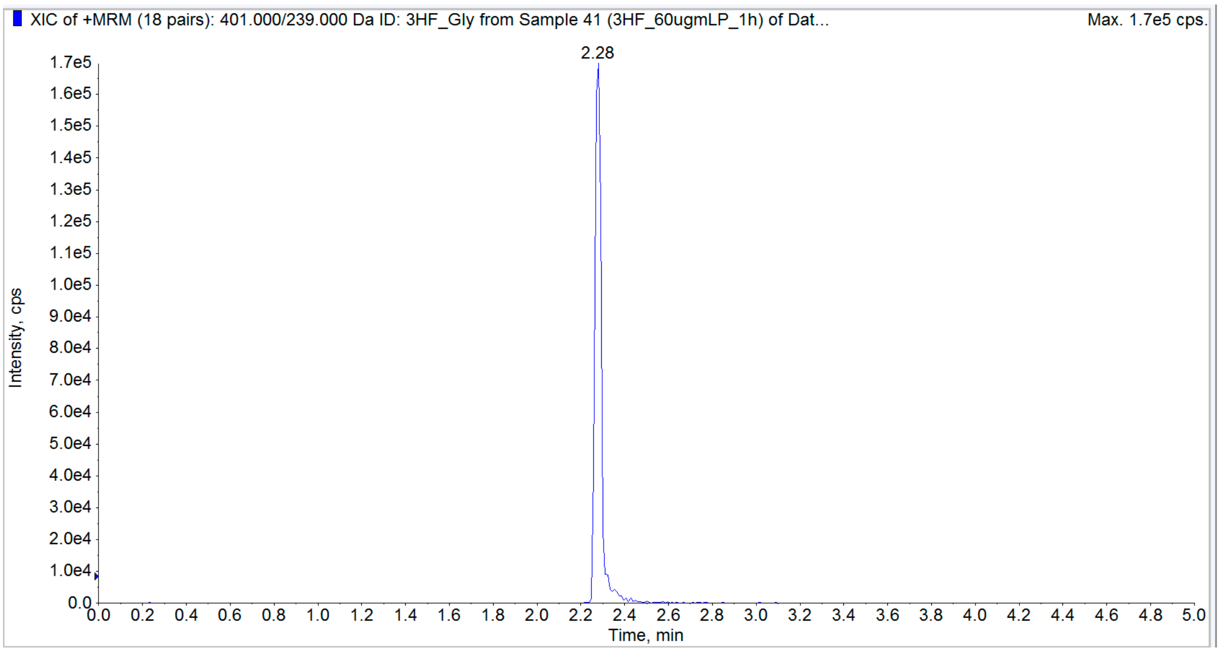

Figure S2. LC-MS Chromatogram of 3HF-glucose (Transition 401/239).

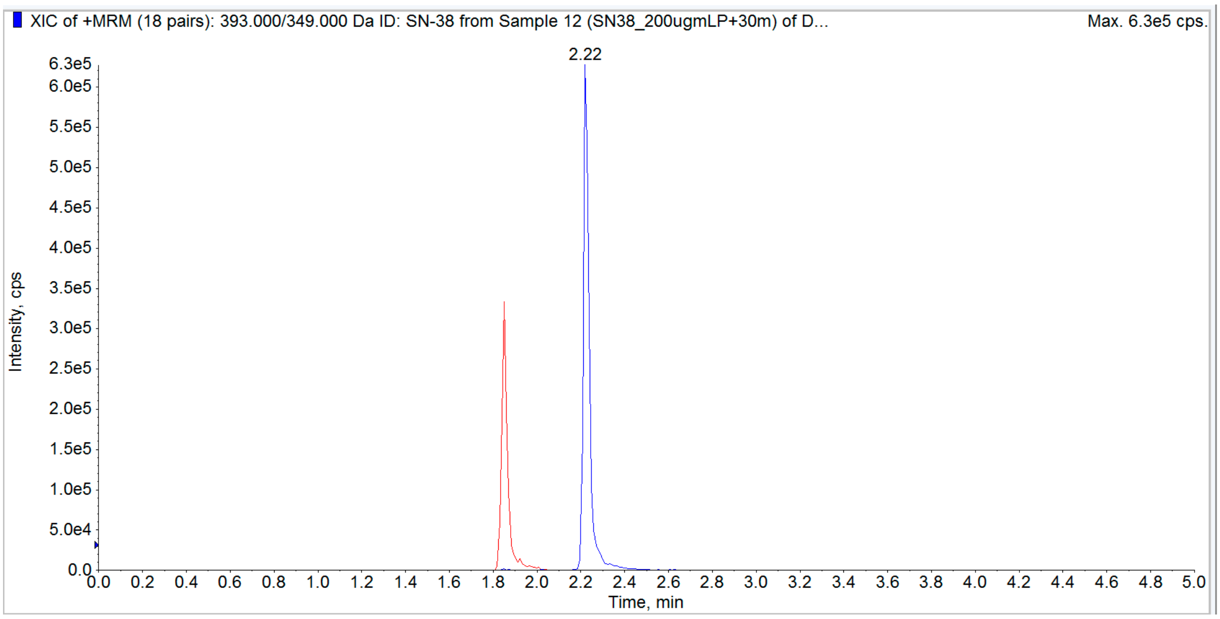

Figure S3. LC-MS Chromatogram of SN-38 and its glucose metabolite (Transition 393/349).

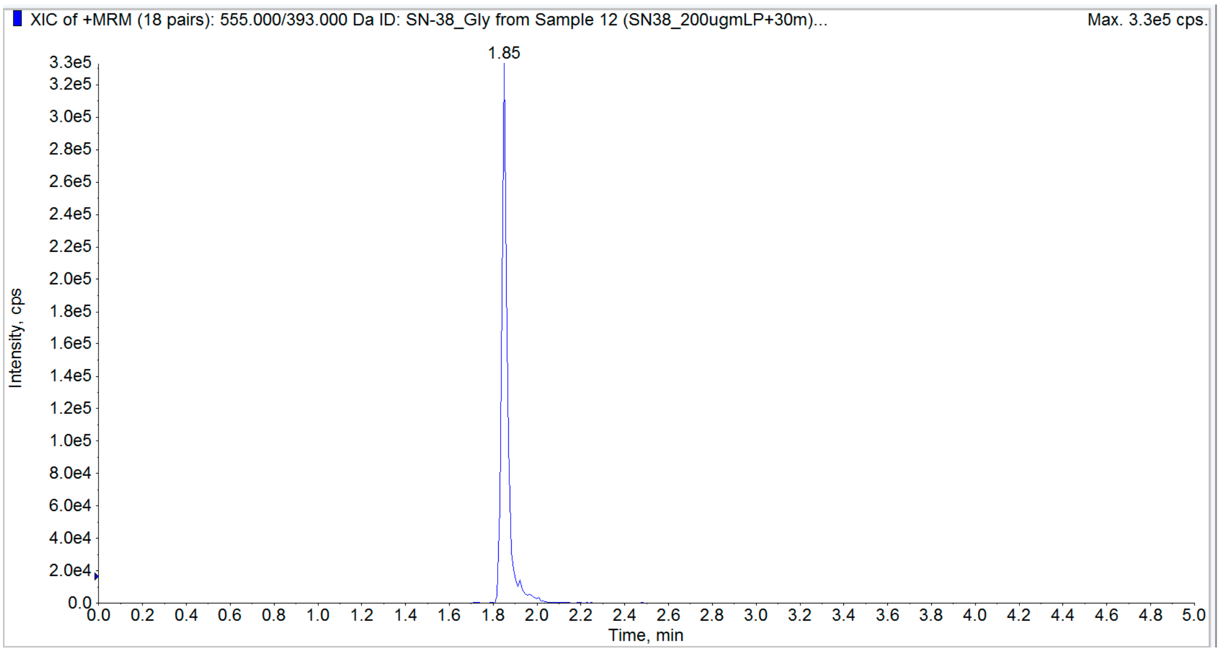

Figure S4. LC-MS Chromatogram of SN-38-glucose (Transition 555/393).

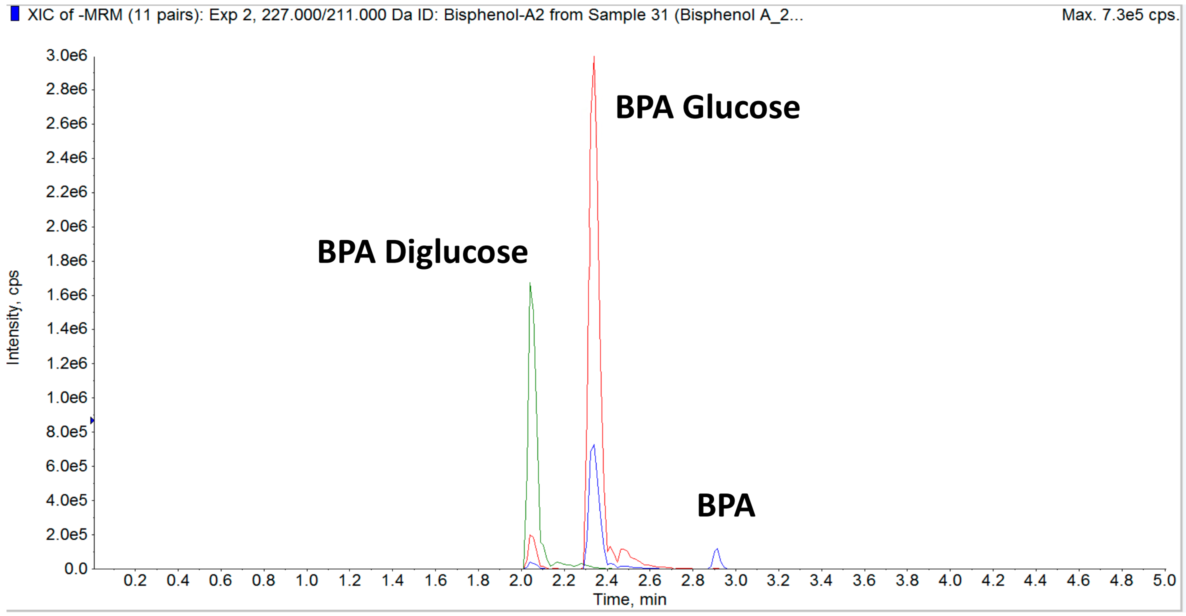

Figure S5. LC-MS Chromatogram of BPA and its glucose metabolites (Transition 227/211).

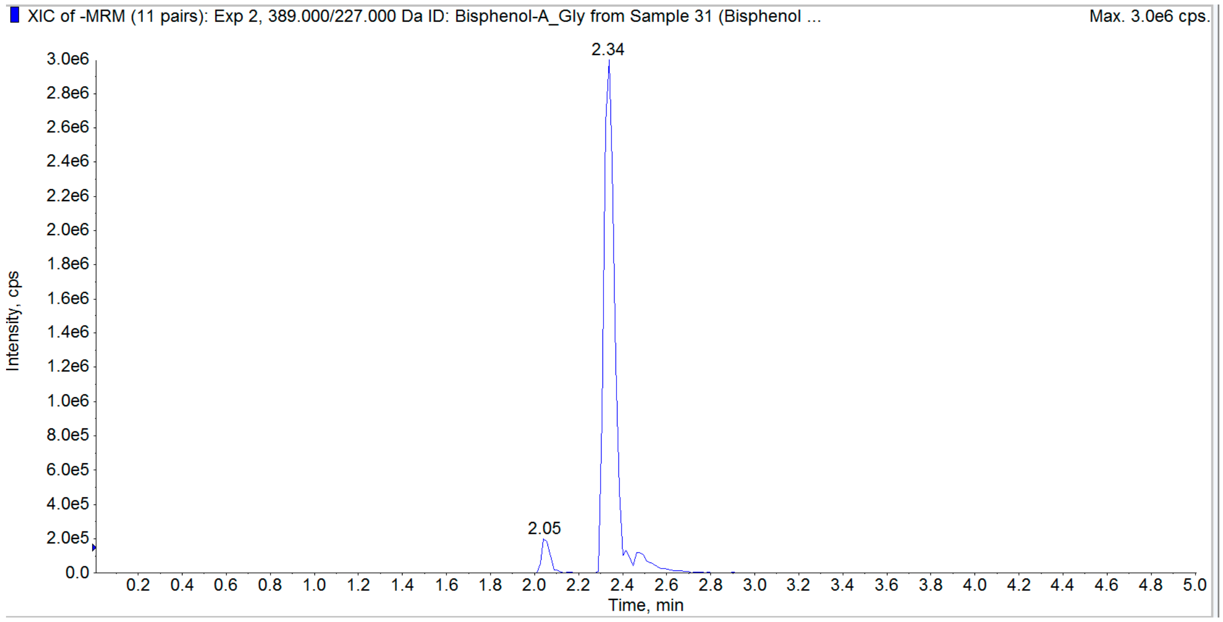

Figure S6. LC-MS Chromatogram of BPA-glucose (Transition 389/227).

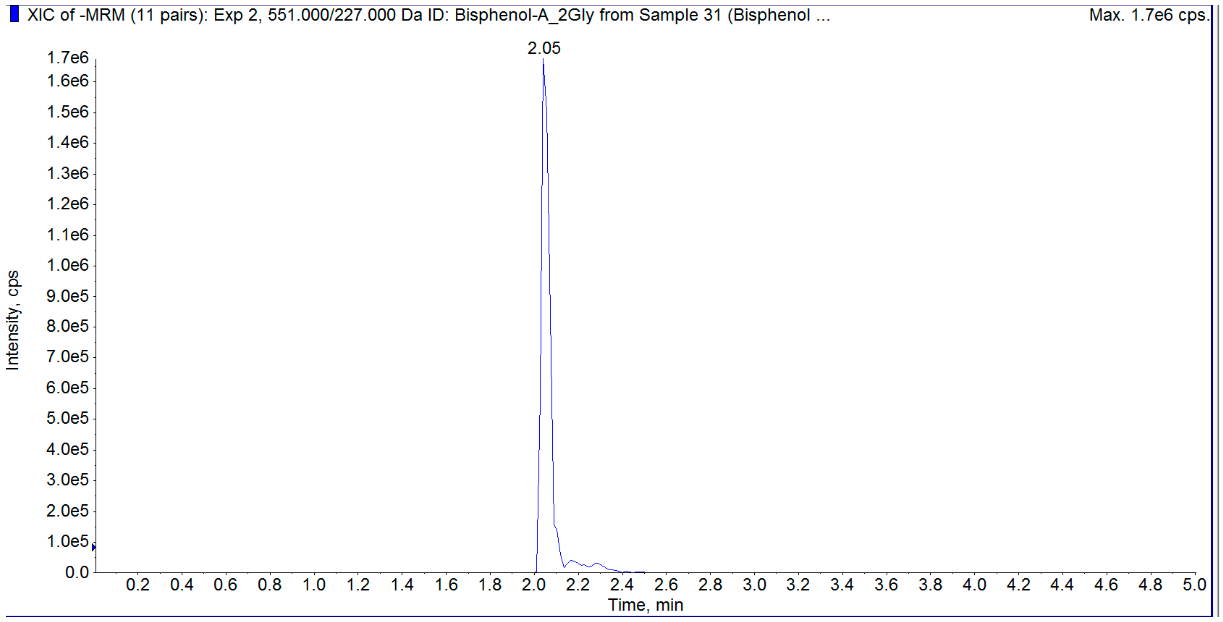

Figure S7. LC-MS Chromatogram of BPA-diglucose (Transition 551/227).
